# A protocol for a randomised double-blind placebo-controlled feasibility study to determine whether the daily consumption of flavonoid-rich pure cocoa has the potential to reduce fatigue in people with relapsing and remitting multiple sclerosis (RRMS)

**DOI:** 10.1186/s40814-018-0230-7

**Published:** 2018-01-23

**Authors:** S. Coe, J. Collett, H. Izadi, D. T. Wade, M. Clegg, J. M. Harrison, E. Buckingham, A. Cavey, G. C. DeLuca, J. Palace, H. Dawes

**Affiliations:** 1 Centre for Movement and Occupational Rehabilitation Sciences, Oxford Institute of Midwifery, Nursing and Allied Health Research, and Oxford Brookes Centre for Nutrition and Health, Oxford Brookes University, Oxford, OX30BP UK; 2 Department of Neurology, Nuffield Department of Clinical Neuroscienes, University of Oxford, Oxford, OX3 9DU UK; 3 School of Engineering, Computing and Mathematics, Faculty of Technology, Design and Environment, Oxford Brookes University, Wheatley Campus, Room R2.32, Oxford, OX33 1HX UK

**Keywords:** Fatigue, Cocoa, Flavonoids, Diet, Multiple sclerosis

## Abstract

**Background:**

Dietary interventions including consumption of flavonoids, plant compounds found in certain foods, may have the ability to improve fatigue. However, to date, no well-designed intervention studies assessing the role of flavonoid consumption for fatigue management in people with MS (pwMS) have been performed. The hypothesis is that the consumption of a flavonoid-rich pure cocoa beverage will reduce fatigue in pwMS. The aim of this study is to determine the feasibility and potential outcome of running a trial to evaluate this hypothesis.

**Methods:**

Using a randomised (1:1) double-blind placebo-controlled feasibility study, 40 men and women (20 in each trial arm) with a recent diagnosis (< 10 years) of relapsing and remitting MS (RRMS) and who are over 18 years of age will be recruited from neurology clinics and throughout the Thames Valley community. During a 6-week nutrition intervention period, participants will consume the cocoa beverage, high flavonoid or low flavonoid content, at breakfast daily. At baseline, demographic factors and disease-related factors will be assessed. Fatigue, activity and quality of life, in addition to other measures, will be taken at three visits (baseline, week 3 and week 6) in a university setting by a researcher blinded to group membership. Feasibility and fidelity will be assessed through recruitment and retention, adherence and a quantitative process evaluation at the end of the trial.

We will describe demographic factors (age, gender, level of education) as well as disease-related factors (disease burden scores, length of time diagnosed with MS) and cognitive assessment, depression and quality of life and general physical activity in order to characterise participants and determine possible mediators to identify the processes by which the intervention may bring about change. Feasibility (recruitment, safety, feasibility of implementation of the intervention and evaluation, protocol adherence and data completion) and potential for benefit (estimates of effect size and variability) will be determined to inform future planned studies. Results will be presented using point estimates, 95% confidence intervals and *p* values. Primary statistical analysis will be on an intention-to-treat basis and will use the complete case data set.

**Discussion:**

We propose that a flavonoid-enriched cocoa beverage for the management of fatigue will be well received by participants. Further, if it is implemented early in the disease course of people diagnosed with RRMS, it will improve mobility and functioning by modifying fatigue.

**Trial registration:**

Registered with ISRCTN Registry. Trial registration No: ISRCTN69897291; Date April 2016

## Background

Fatigue is one of the most common and debilitating symptoms experienced in people with MS (pwMS), and it is thought that as many as 53–90% of pwMS suffer from fatigue [1].

Currently, there are no highly effective treatments for fatigue in MS; however, simple lifestyle interventions including diet approaches may be beneficial for reducing the extent of fatigue [2].

The physiological causes of fatigue are complex, including neural, inflammatory and metabolic mechanisms, and can result in further changes including increased cytokine production, oxidative stress and altered glutamate/glutathione levels in the brain, all thought to contribute to fatigue [3–7]. Certain compounds in foods called flavonoids may be used to exert drug-like effects in reducing the severity of fatigue by tackling these mechanisms, and these foods are becoming increasingly popular for their alleged ubiquitous benefits on health, the ageing process and neurological function [8].

Dark chocolate containing 70% or more cocoa solids is well known for its high antioxidant and flavonoid content. Over a 4-week period, dark chocolate consumption has been shown to improve fatigue in those with chronic fatigue syndrome (CFS) which could part due to the improvements in immune function that is often seen with the condition [9]. Also, results from a small randomised controlled pilot study and using a short-term cocoa intervention suggested an increase in sleep quality and reduction in fatigue [10].

Alterations in inflammatory and immunological function have been shown to precede relapses in those with relapsing and remitting MS (RRMS) [11] and have been thought to contribute to the early symptoms of fatigue [12–14]. This has therefore raised the question as to whether alleviating these markers may improve the fatigue experienced early on in those with the disease and improved mobility and physical activity [15, 16].

This study sets out to determine the potential benefit and feasibility of implementing a flavonoid dietary intervention that was co-designed with pwMS. We propose that a single drink of flavonoid-rich cocoa taken each morning over a 6-week period will be feasible and acceptable for pwMS. We will also estimate the variation and change in fatigue to allow for a power calculation to be made for a larger trial. In addition, there is a process evaluation of both quantitative and qualitative components. The study uses a randomised, parallel group controlled design where participants will either be supplied with cocoa rich in flavonoids or a low-flavonoid cocoa drink that looks and tastes the same.

### Objectives

To determine whether the current study design is feasible to undertake, and to estimate the potential benefit and number of participants needed for a phase III trial, the following key objectives will be assessed from results of this study:

1. The acceptability of the study design and dietary intervention to participants
2. Recruitment rate and reasons for non-recruitment
3. Follow-up rate and reasons for loss to follow-up
4. Level of adherence to the protocol, including the process of randomisation
5. A parallel process evaluation to explore participant acceptance, potential concerns and overall process flow of the proposed intervention
6. Data acquisition rate, amount of missing data and reasons for loss
7. To determine the variability and effect size of the outcome measures, especially on fatigue.

## Methods

### Setting

All testing will take place at the Oxford Brookes University (OBU), Oxford, UK, except for home visits in which participants have the option for researchers to visit their homes to collect measures. The intervention will be self-directed and take place in the home of each participant. Testing will take place in the morning between 7.30 and 10 a.m.

### Recruitment

PwMS will be recruited from the Oxford University Hospitals National Health Service (NHS) Trust (John Radcliffe Hospital site), Milton Keynes Hospital, Royal Berkshire Hospital or Buckingham Hospital by nurses and clinicians at each site who work with MS patients, through a list of those who have consented to being contacted about clinical trials. Potentially eligible participants who are interested in the study will have their contact details collected by the lead researcher who will provide further information. Local MS Society branches will also be made aware of the trial and given contact details, and an advertisement for the study along with the participant information sheet will be made available on the MS Society website. Individuals will then be able to self-refer to the study.

### Trial design

**Fig. 1:**
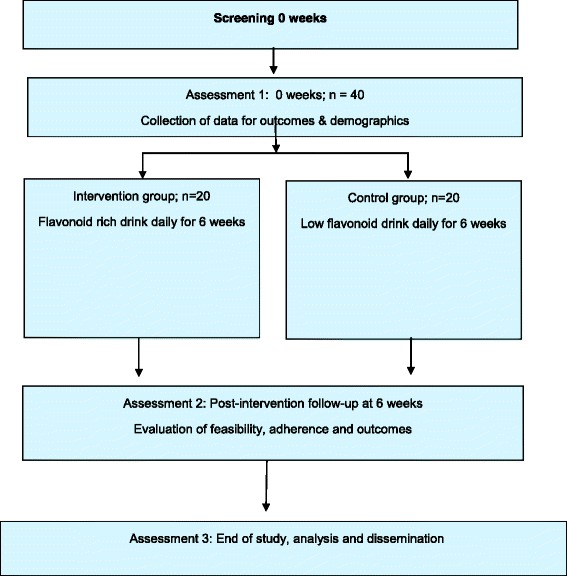
Trial schema and participant flow diagram

This is a mixed methodology, randomised double-blind placebo-controlled feasibility trial and process evaluation. As a feasibility trial, aspects of feasibility will be determined and estimates of effect size and measured variability will be calculated to inform future planned studies. The intervention will last a total of 6 weeks. Participants will be assessed at baseline on enrolment into the trial. Randomisation to allocate participants to either intervention or control group (1:1) will be performed immediately following the baseline assessment, and intervention delivery will begin directly after the baseline assessment. Participants will then be reassessed at 3 and 6 weeks (assessments 2 and 3, respectively) after the baseline. At assessment 2, the lead researcher accompanied by one of the research team will offer to travel to the home of the participants to take measures, if they so wish (Fig. 1). This is to reduce patient burden with the aim to increase recruitment and retention rate.

### Participants

Participants will be considered eligible for the study if they present with the following criteria:

1. Aged 18 years or more with a new clinical diagnosis of RRMS within the past 10 years
2. Those who are treatment naïve or taking first-line disease-modifying treatments (DMTs) (glatiramer acetate, interferon beta, teriflunomide and dimethyl fumarate, but also alemtuzumab and Tysabri if used as first-line treatments)
3. No relapse or sudden change in their MS symptoms within the previous 3 months
4. Do not have contraindications to providing a blood sample
5. No contraindications to tolerating the cocoa drink
6. A fatigue measure of greater than 4 on the Fatigue Severity Scale (FSS) [17]
7. No other conditions that may be associated with fatigue, e.g. anaemia
8. No clinical diagnosis of depression
9. An Expanded Disability Status Scale (EDSS) score of < 4.5
10. Sufficient mental capacity to consent as determined by the researchers
11. Able to walk with or without a walker for at least 16 m
12. No condition affecting the central nervous system other than MS (however, migraine is allowed)
13. Not pregnant or lactating
14. No objection to the researchers contacting their general practitioner and neurologist

Participants must also not be on any other clinical trial during the study.

### Consent

Participants will be emailed or personally administered an information sheet by clinicians at one of the hospital sites or by a member of the research team and given at least 24 h to consider the information. An eligibility check will be performed over the telephone by one of the researchers. During the telephone call and at the baseline assessment, potential participants will be allowed to ask questions if they wish to participate in the study, and enrolment into the trial will be immediately allocated. Informed consent will be obtained by members of the research team at the baseline assessment, who will be trained in taking consent and in good clinical practice. Recruitment began in May 2016.

Withdrawal of consent will have no detrimental impact on current and future treatment, and those taking consent will ensure that participants fully understand this. If a participant was to lose his/her capacity to consent during the trial, the participant will be withdrawn, no further procedures will be carried out on them and no new personal data will be collected. Data that has already been collected in relation to the participant may be retained and used for the purposes for which consent has already been given, provided they are effectively anonymised and no longer identifiable to the research team.

### Retention

We will make every effort to reduce loss to follow-up. One primary contact within each centre will contact all participants and act as liaison with the blinded assessor. The primary contact person will obtain information about the participants’ preferred method of contact (text, telephone, email and letter) and contact them using this preferred method when possible. They will also ask the participant for details of a nominated person who can be contacted if the team is unable to make contact with them, and we will retain details of the participant’s general practitioner. Contact details will be updated if applicable at each assessment. All appointments will be made at the research participant’s convenience wherever possible. If an appointment is missed we will try to rearrange this on two more occasions.

### Screening

A screening log will be kept at OBU where details of numbers of people who were approached about the trial, eligibility and whether consent to be contacted was given or declined will be recorded. A log of any individuals, who declined at the initial consent stage, or consented but failed screening, will also be kept. A case report form (CRF) will be completed for all consented individuals, which will include details of nominated persons (e.g. mother or friend) to facilitate participant follow-up.

### Outcomes

The primary aim of this study is to assess feasibility of the dietary intervention in terms of adherence, safety and process. This will be achieved through the documentation of adherence and any adverse events (AEs), as well as any health-related changes such as gastrointestinal disturbances that may occur during the intervention period. Duration of participation and dropout from the intervention will also be recorded. In order to assess benefits, a range of outcomes will be obtained. Outcome measures include

1. Levels of physical activity: wearable activity monitors (worn like a watch) [18] and the Physical Activity Scale for the Elderly (PASE) questionnaire [19]
2. Dietary patterns assessed by a nutritionist: three 24-h dietary records [20]
3. Fatigue levels: FSS, Neuro-QOL [21], and during the intervention, a Numerical Rating Scale (NRS) scored from 1 to 10, three times daily which classifies no fatigue as a 1 and very fatigued as a 10.
4. Fatigability: performance on 6-min walk test (physical) [22] and the Adult Memory and Information Processing Battery (AMIBP; mental) [23]
5. Blood markers of inflammation: TNF-alpha, reduced glutathione, a marker of antioxidant status and lipid peroxidation
6. Health descriptives: EQ5D5L [24] and the Preference-Based Multiple Sclerosis Index (PBMSI) [25], basic health questionnaire, Barthel ADL Index (BI) [26] and Hospital Anxiety and Depression Scale (HADS) [27]

Demographic factors and background factors will be assessed at baseline: medication, age, gender, level of education, type of MS, duration of disease, height, weight and leg length will be assessed at baseline. Participants will be stratified for gender and whether they are on first-line disease-modifying treatments or not.

Table 1 summarises the questionnaires that will be administered throughout the trial and at which visits these measures will be collected.

**Table 1:** Outcome measures

Domain to be measured	Outcome measure(s)	Time required	When
Measures of overall health and questionnaires			
1. Health questionnaire and health assessment	General medicine use/health problems, etc.	10 min	1
2. Global fatigue	Neuro quality of life	5 min	1, 2, 3
3. Limitations in daily activity	Barthel Activities in Daily Living Index	5 min	1
4. Quality of life measure	EuroQOL 5D5L and Problem Based Multiple Sclerosis Index	5 min	1, 2, 3
5. Dietary recall	Monitor diet patterns	10 min	1, 2, 3
6. Consent	Verbal followed by written	10 min	1
8. Speed of information processing	The Adult Memory and Information Processing Battery	5 min	1, 2, 3
9. Depression	Hospital Anxiety and Depression Scale	5 min	1, 2, 3
10. Physical activity	Physical Activity Survey for the Elderly	10 min	1
11. Fatigue	Fatigue Severity Scale	5 min	1
Measures of activity and fatigue			
11. 6-min walk	Fatigue	6 min	1, 3
12. Accelerometer	Activity	2 weeks and 3 days throughout intervention	–
13. Numerical Rating Scale for fatigue	Fatigue	3 times daily for 6 weeks	–
Measures of plasma markers			
14. Venous blood samples	Inflammation, oxidative stress, antioxidant capacity of plasma	5 min	1, 2, 3

1 = baseline assessment; 2 = assessment 2 (3 weeks); 3 = assessment 3 (6 weeks)

### Intervention

#### Cocoa

Participants will consume the drink (intervention or control) in their homes, after a 10-h overnight fast at the same time each morning. After the consumption of the drink (18 g of cocoa in 200 ml heated rice milk), they will wait an additional 30-min period before consuming any other food or beverage. They will be instructed to take their normal medication and to follow their diet as usual. On the days when assessments take place at the university, participants will arrive fasted and will therefore not consume the cocoa that day. The cocoa drinks (intervention and control) are designed to differ only in flavonoid content (low {19 mg cocoa flavonoids} versus high {200 mg cocoa flavonoids} flavonoid content). The flavonoid-rich pure cocoa drink will be matched to the control drink for macronutrients, theobromine and caffeine and will be as identical as possible in appearance and taste to ensure double blinding. Cocoa powder will be provided in air-tight individual sachets, and participants will receive 21 packets at the baseline visit and again at assessment 2; the contents will need to be added to a mug, and heated rice milk will be added prior to consumption. Instructions on preparation will be provided to ensure all participants follow the same protocol. Unused cocoa content will be saved and collected by the researcher.

### Fatigue

Fatigue will be measured at baseline using the FSS. If participant scores a 4 or above and thus fulfils the criteria to take part in the trial, he or she will then wait 3 days before beginning the intervention. On the fourth day, the participant will begin consuming the coca drink. Directly after the consumption of the drink, participants will rate on an NRS their level of fatigue. This will be followed by two additional measures recorded at 3 p.m. and 8 p.m., daily for the 6 weeks. They will be asked to reply to the text message with a score of fatigue from 1 to 10, 1 being no fatigue and 10 being very fatigued. Smart phones will be provided to those who do not have them and collected after the trial termination, and ease and acceptability of phone use will be documented. Walking endurance will be measured a total of two times (baseline and assessment 3) using a 6-min walk test [22], and sensors will be worn to measure gait during the walk. Gait measurements will be collected via an inertial measurement unit (IMU) [28, 29] attached to the lower spine (lumbar 4 region) by double-sided adhesive tape. Participants are instructed to walk over a 14-m walkway free of obstacles at their self-selected walking speed. Walking speed will be derived from IMU data by using well-established validated algorithms [28, 29].

### Blood measures

Participants will have fasting blood samples taken by a trained phlebotomist a total of three times throughout the trial. Blood samples will be collected at OBU or at the home of the participant (assessment 2). Blood collection will be in the morning when the participant is fasted, time will be recorded at the first collection and collection for subsequent assessments will be taken within an hour of this time. Blood will be labelled and frozen for future analysis following standard operating procedures (SOPs) for storage of plasma at OBU. Fasting plasma glutathione (oxidised and reduced) will serve as a measure of adherence to the consumption of the drink throughout the trial. Changes in levels of the inflammatory marker TNF-alpha (ELISA assay kit) and lipid peroxidation (MCA reaction assay) will also be measured as markers of inflammation and oxidative stress, respectively, at the same time points. Assays for these individual markers will be purchased from Sigma-Aldrich (UK) and will be analysed using facilities available at OBU. Outcome measures will be determined from venous blood (total at each assessment no more than 50 ml) samples taken three times throughout the intervention—baseline assessments 1 and 2, after an overnight (10–12-h) fast. These measures will determine the change in metabolic parameters throughout the trial and will also allow for adverse changes made possible due to the consumption of the control drink. In addition, compliance and willingness to have blood measures taken will be documented.

### Activity

Accelerometers will be worn for 3 days before starting the cocoa and then for the third and sixth weeks (7 days each, 17 days in total) after starting. They will sample data at 25 Hz [18], and data will be averaged for each day. Participants will be reminded to initiate wearing the accelerometers by the lead researcher via text messages. The acceptance and adherence to wearing the accelerometers during this time will be assessed at the final assessment.

### Diet

A total of three 24-h dietary recalls will be administered to provide information about the diet of each participant and to determine any changes that may take place throughout the study. This method is less burdensome for patients than a 7-day food record diary yet will still provide an overall pattern in dietary behaviour throughout the study. Total energy intake is a potential confounder of the study and will therefore be corrected for in the analysis. We advise participants not to change their habitual diet and exercise regimes during the trial.

### Sample size

A sample size of 40 pwMS was deemed appropriate to address feasibility and determine variability of outcome measures [30–32] for a follow-on trial based on a 12-month recruitment period. Approximately five potentially eligible patients with recently diagnosed RRMS attend the clinic at the hospital per week, of which three will potentially fit the inclusion criteria of the study. Considering a recruitment rate of approximately 1.5 patients per week (50%), this would enable a minimum sample of 40 pwMS. The sample size of ≥ 30 also allows for assumptions from the central limit theorem in relation to representation within this group of RRMS with fatigue and should be easily recruitable within the timeframe.

### Data management

All work undertaken as part of this trial will comply with the Research Governance Framework for Health and Social Care UK and the OBU Research Governance Framework. All participant identification and referral procedures as well as procedures for data storage, processing and management will comply with the Data Protection Act 1998. All assessment data will be collected face-to-face using paper data collection forms. AE forms will be collected in person by one of the research team at OBU named in the delegation log. The retention period complies with guidelines set out by the OBU Research Governance Framework. Any individual who discontinues or deviates from the intervention will still have their data included in the data analysis.

### Safety monitoring

All chemicals required for assays will be purchased from Sigma-Aldrich, UK. SOPs have been approved by OBU and will be utilised for conducting all outcome assessments.

There are no expected serious adverse events (SAEs) in this study. The proposed procedures carry minimal risk to participants, and their care and comfort will be ensured throughout. This research will be performed in accordance with the International Conference on Harmonisation-Good Clinical Practice (ICH-GCP) standards. National Research Ethics Service ethical approval will be sought and confirmed before the start of the trial. The study will be overseen by a steering committee who will act as the data-monitoring committee to consider AEs and/or lack of adherence to the protocol (objection to having blood samples taken, lack of tolerance to the control/test drink).

### Randomisation

After recruitment, confirmation of eligibility, giving consent and collection of baseline data, patients will be registered and then randomised into one of the two groups, either the control or the test cocoa group. Participants recruited to the study will be randomly allocated the next available study number by the blinded assessor, with 20 participants in each group. The unblinded researcher will randomise participants into the control or intervention group using a computer-generated randomisation groups stratified for gender and DMTs.

### Blinding

This is a double-blind trial. One of the researchers on the trial will have access to the randomisation list, in which she will allocate participants randomly to either group. Each cocoa pack will contain a unique three-digit code, which will be determined by the unblinded researcher who will be able to trace the codes back to the cocoa group. All data collection will be conducted by a team of blinded assessors specifically trained in the methodology utilised for the collection of physical activity and functional assessments. Records of incidents where blinding is broken will be kept. All researchers who have contact with the participants will be blinded, including the main researcher on the trial. The main researcher and the statistician will remain blinded until after data analysis. Also, at the end of the intervention but before disclosure, participants will report their opinions of which group they believe they have been allocated to. A SAE could result in unblinding; however, this is unlikely as the principal investigator (PI) and the consultant medic on the trial will assess all AEs and SAEs and will make a decision of the action.

### Process evaluation

Upon exiting the study at assessment 3, each patient will be interviewed about queries regarding the intervention process, ease of adherence, tolerance and acceptability of the flavonoid drink and collection of outcome measures, and asked about any other issues or concerns arising from the trial. Participants will be asked their opinion on the importance of the research question proposed to inform future trials. We will also attempt to contact any participants who drop out of the intervention to ascertain reasons for discontinuing. Topics will include challenges to intervention delivery, perceived successes, barriers to implementation and suggestions on how to improve the intervention process. All participants who complete the trial only will be interviewed.

We will employ standard thematic analysis techniques. The transcripts of interviews will be closely examined to identify themes and categories. Codes will be applied to these broad themes, which will then be broken down further into sub-codes. Agreement on concepts and coding will be sought between members of the research team to ensure reliability. We will identify commonly expressed themes as well as unusual cases. A proportion of the data (20%) will be coded by two different team members to check on reliability of the coding scheme. The process of data collection and data analysis will be iterative, so that new themes emerge, and we will incorporate them into subsequent interview schedules. Process evaluation will include frequencies for adhering to the intervention, session content and progression which will be analysed descriptively with confidence intervals and regression where possible.

### Ethical and regulatory considerations

The trial has received approval from an NHS Research Ethics Committee (NHS REC, 16/WM/0134) for approval. Site-specific approvals for NHS PIC sites have been obtained. This is consistent with the requirements of OBU, who has agreed to act as the trial sponsor. All protocol and trial amendments (both minor and major) will be submitted through the ethics committee and, once approved, will be communicated to the sponsor and funding body and to the steering committee. The confidentiality of participants will be preserved in accordance with the Data Protection Act 1998. All participants will be allocated a unique identifier, and all trial data collected will be held in a linked anonymised form. Identifiable information will be stored separately from trial data. The randomisation will be held by a member of the research team who will oversee the trial but will not be involved in data collection or analysis. Group allocation will be referred by this same member and will ensure double blinding.

### Dissemination policy

Results of this trial will be reported in the first instance to the funders and then communicated to participants and relevant health professionals in a series of open access publications within 9 months of the end of the data collection. Authorship will follow the trial publication policy that has been developed based on *British Medical Journal* rules on authorship and contributorship. All publications will be deposited in the university open access forum for publications and, as much as possible, will be published in open access journals and will be open access on demand. The *BMJ* guidelines on writing authorship will be adhered to, and all members on the trial steering committee will sign and agree.

### Discontinuing

Participants will be advised to discontinue the trial if they find consuming the cocoa unacceptable or if consuming the cocoa is associated with in any related or unrelated AEs. Discontinuation of the trial will ultimately be decided by the PI and consultant medic.

#### Auditing

The trial is open to auditing either through NHS governance or through the university’s Centre for Movement, Occupational and Rehabilitation Sciences governance committee.

### Statistical methods

We will report summary statistics for demographics and outcome data of the study participants. The assessment of mediators is important to be able to identify the processes by which the intervention may bring about change. The proportion of adherence to the intervention will be reported descriptively with 95% confidence intervals. Graphical illustration will be used to check distributions of outcome data. As an exploratory trial, the primary outcome measures will be feasibility and acceptability. Results will be presented using point estimates, 95% confidence intervals and *p* values. The primary statistical analysis will be on an intention-to-treat basis, and the primary analysis will use the complete case data set. Exploratory analyses will be used to investigate the impact of individual demographic factors as well as theoretical mediators (i.e. disease/fatigue severity) on the intervention effect using appropriate correlation coefficients.

#### Feasibility

Feasibility will be analysed through evaluation of eligibility, recruitment and retention (in line with CONSORT extension recommendations). Adherence to intervention will also be assessed. Completeness of outcome measures will be reported, and 80% would be considered appropriate for each measure. Feasibility will be determined and estimates of effect size and outcome variability will be calculated to inform future planned studies.

#### Eligibility

Eligibility will be assessed through screening logs. We will report on the proportion of screened participants who were deemed eligible for the study, and summarise the reasons for ineligibility.

#### Recruitment

Recruitment will be evaluated based on the final number of participants who were successfully consented and randomised.

#### Retention (i.e. assessments)

Retention will be measured by evaluating the proportion of participants who were lost to follow-up.

#### Adherence

Successful adherence to the intervention will be defined as at least 75% of the participants having completed cocoa consumption [33]. We will assess discontinuation of the intervention. Further aspects of adherence will be measured by the percentage of fatigue texts completed by participants and those who wore the accelerometer-axivity watches. If the proportion is less than this but greater than 65%, we will consider adjusting the intervention to increase this in future investigations. An adherence rate lower than this would require substantial changes to the intervention and therefore require further piloting.

#### Acceptability

The acceptability of the intervention will be measured through the end-of-study interview, which uses questions to measure the extent to which participants found the intervention and materials useful, enjoyable and beneficial.

Exploratory analyses will be used to investigate the impact of individual demographic factors as well as theoretical mediators on the short-term benefit of the intervention, using appropriate correlation coefficients.

A generalised linear model analysis will investigate whether fatigue differed between the treatment arms. This will be extended to joint modelling of both fatigue and physical functioning if there is a significant difference in the reported fatigue. Data may be transformed to improve model fit, or different regression approaches used (e.g. negative binomial or Poisson regression).

### Trial sponsorship

OBU is the sponsor for the trial.

### Funding

Funding for the trial was granted from the Multiple Sclerosis Society.

### Trial status

The first participant was consented on the 15th of June 2016. The trial is currently recruiting until the 31st of August 2017.

## Discussion

This study sets out to estimate the extent of the impact and feasibility of implementing flavonoids as an intervention for fatigue management in PwMS. Patients, carers and clinicians agree that fatigue is one of the most debilitating symptoms, and while there is no effective drug therapy to treat fatigue in this group and no clear understanding of the underpinning mechanism, diet offers a logical method to act on some of the suggested possible mechanisms [3–7] and reduce fatigue severity and improve activity and mobility in PwMS. Previous studies have been mostly observational cross-sectional studies with inconclusive results for determining the best nutritional interventions for symptom management in MS. Unlike in the field of physiotherapy/exercise science where there are a number of interventional studies and burgeoning evidence of optimal and safe exercise interventions to support symptom management, there is a paucity of good quality trials investigating dietary interventions and extremely limited evidence. There are also no other methods described for controlling and implementing diet so this study will provide a framework for dietary intervention and monitoring in PwMS and other groups. This study is therefore designed not only to pave way for a fully powered, targeted, well-controlled, cost-effective intervention study with minimal risks but also to develop methodology for implementing dietary trials in people with MS and other neurological conditions. The current application also focuses on the priority to refine existing treatments from other conditions as treatments for MS and is based on successful research on fatigue in people with CFS. The results from this study will be generalisable to those with RRMS and high levels of fatigue, and therefore, future trials will need to explore generalisability to other types of MS and for those with lower fatigue.

There is limited infrastructure in place to support the development of high-quality research and evaluation of nutritional interventions and exposures. This project also brings together key stakeholders enabling an interdisciplinary team involving clinicians, researchers and PwMS to ensure the trial is performed and disseminated to the best of its ability, leading to further collaborations and funding. This feasibility trial is utilising a range of outcome measures in order to gather all relevant and comprehensive data to produce results that inform a follow-on trial. This project takes a multi-pronged approach to measuring fatigue, understanding the basis of differences in fatigability and assessing potential interventions.

### Risk management

The addition of a cocoa beverage to the diet of pwMS may cause concern for those taking part due to the added calories. By consuming cocoa in a concentrated drink form, the total calorie content is reduced compared to if a chocolate bar was consumed and, therefore, weight gain will not be of concern and we are recording weight at the beginning and end of the intervention. There has also been reporting of a reduction in appetite after dark cocoa consumption, and this will be monitored during the 24-h recall at each visit. Research, to date, has not reported any potential side effects of cocoa or any allergies associated with its consumption. The flavonoid-rich pure cocoa drink will be matched to the control drink for macronutrients, theobromine and caffeine and will be at safe levels of intake. There is a risk of participants burning themselves adding the rice milk to the drink; however, people routinely make hot drinks in their homes and, therefore, this risk is not above the risks involved in daily activity. Participants will be asked about their habitual diet using a 24-h recall at baseline, assessment 1 and assessment 2. This will monitor any significant or potentially harmful changes to the diet that are taking place throughout the trial.

It is possible that a participant could trip or fall during the 6-min walk test. However, the team routinely administers walking tests with people with neurological conditions including people with MS (pwMS) with no AEs. Questionnaires used in the study may cause mild distress to participants; however, they are all commonly used questionnaires. Should a participant become distressed, we would stop the assessment until and if the participant feels able to continue.

The blood samples will be taken with minimal discomfort. Some individuals may experience an increased sensitivity on the forearm, but this will disappear in 1 to 2 days and should not affect a participant’s work. Minor risks are associated with sampling blood, including fainting, infection and bruising. Sampling will be performed by a trained phlebotomist, thus reducing the risks significantly. A first aid provider will be available at all times during testing. There will be a designated clean area where blood will be taken. Clinical waste procedures will be followed at all times.

Attending baseline and final assessment at OBU will pose some risk in travelling to the site. However, participants will be provided with transport and any other assistance that they need in order to feel comfortable and safe. To reduce participant burden, they will have the option of having the researchers come to their homes in order to take measures at assessment 2. The OBU lone working policy will be applied. Researcher will risk assess on arrival at a person’s home and terminate the visit if there are any causes for concern. The researcher will establish a contact point with the research team at OBU so that researcher’s whereabouts are known. A mobile phone text message will be sent to confirm safe arrival at the participant’s home and on leaving the home. Researchers will always ensure that participants are fully aware of the time and date that they will be attending a home visit.

### Feasibility

We will consider the acceptability of the intervention to participants, the ability of centres to recruit participants, the willingness of participants to be randomised, the number of eligible participants, the acceptability of the measures and the time taken to collect data.

From a design perspective, we have explicitly considered the burden with respect to timings of assessments and intervention sessions. AEs will be systematically documented, and if participants choose to discontinue the intervention, sites are encouraged to continue with outcome assessments wherever possible. Finally, data monitoring will be extensive to ensure minimisation of missing forms or data, and analysis will consider imputation methods as required. The data gathered here will inform the design (including sample size calculations) and delivery of a confirmatory phase III trial. In order to ensure repeatability in confirmatory trials, reporting will follow the template for intervention description and replication (TIDieR) reporting guidelines for description of interventions, and the Consolidated Standards of Reporting Trials (CONSORT) extension for non-pharmacological interventions. The possibility of refining measures will be considered by the research team, and key features of the feasibility trial will be considered for future work.

The end of the trial will be considered as the date on which the last participant has completed his or her final assessment.

## Abbreviations

AE: Adverse event
AMIPB: Adult Memory and Information Processing Battery
BI: Barthel ADL Index
CFS: Chronic fatigue syndrome
CRF: Case report form
EDSS: Expanded Disability Score Scale
FSS: Fatigue Severity Scale
HADS: Hospital Anxiety and Depression Scale
ICH-GCP: International Conference on Harmonisation-Good Clinical Practice
IMU: Inertial measurement unit
NRS: Numerical Rating Scale
OBU: Oxford Brookes University
PASE: Physical Activity Scale for the Elderly
PBMSI: Preference-Based Multiple Sclerosis Index
pwMS: People with multiple sclerosis
RRMS: Relapsing and remitting MS
SAEs: Serious adverse events
SOPs: Standard operating procedures
